# Clinical Status of Cardiac Rehabilitation Manners and Models

**DOI:** 10.1155/2022/9554984

**Published:** 2022-06-27

**Authors:** Wei Wei, Jingjie Zhao, Lingzhang Meng, Xi Wang, Hongdi Wei, Keji Nong, Jiahao Li, Zechen Wang, Jiajia Shen, Siyuan He, Lihua Yang

**Affiliations:** ^1^Cardiovascular Medicine Department, Jiangbin Hospital of Guangxi Zhuang Autonomous Region, Nanning, China; ^2^Life Science and Clinical Research Center, The Affiliated Hospital of Youjiang Medical University for Nationalities, Guangxi Zhuang Autonomous Region, Baise, China; ^3^Center for Systemic Inflammation Research (CSIR), School of Preclinical Medicine, Youjiang Medical University for Nationalities, Guangxi Zhuang Autonomous Region, Baise City, China

## Abstract

Cardiac rehabilitation, which combines cardiology and preventive medicine, is an important part of treatment for cardiovascular diseases. Systematically, cardiac rehabilitation, including simultaneously inhibiting endothelial injury and promoting endothelial repair, is beneficial for physical and mental recovery and reduces the risks of recurrence and death in patients with cardiovascular diseases. Cardiac rehabilitation has developed rapidly in the last 50 years. A preliminary system for cardiac rehabilitation has been developed in China. The present article mainly focuses on the progress of cardiac rehabilitation from the aspects of goals, measures, and modes of research in the current scenario.

## 1. Introduction

Cardiac rehabilitation has a history of more than 200 years and has rapidly developed for nearly half a century. At present, approximately 54.7% of countries worldwide, mainly including the middle and high-income countries, have already started cardiac rehabilitation [1]. In recent years, its role and position in cardiovascular medicine in China has been gradually accepted. Modern cardiac rehabilitation integrates several disciplines, including cardiovascular medicine, sports medicine, nutrition, psychology, behavioral medicine, and preventive medicine. The aim of cardiac rehabilitation is to achieve recovery as soon as possible, reduce the recurrence and mortality of cardiovascular diseases, and reduce medical and healthcare expenditure. Cardiac rehabilitation has been included in the clinical medical care quality evaluation system in the United States; medical insurance in Germany also requires patients to undergo cardiac rehabilitation [1]. Cardiac rehabilitation has been listed as a Class I recommendation for the prevention and treatment of cardiovascular diseases by several European and American cardiology academic organizations [2]. The research progress in cardiac rehabilitation is briefly described in this review.

## 2. Means of Cardiac Rehabilitation

In 2007, the American Association of Cardiovascular and Pulmonary Rehabilitation/American Heart Association (AACVPR/AHA) defined cardiac rehabilitation as a comprehensive and coordinated long-term plan that includes medical care evaluation, exercise prescription, correction of cardiovascular risk factors, education, consultation, and behavioral intervention [3]. At the beginning of the 21st century, Professor Hu Dayi [3] concretized the means of cardiac rehabilitation into five prescriptions: medication, exercise, psychological, nutrition, and smoking cessation. The clinical function of cardiac rehabilitation mainly lies in the prevention and treatment of the risk factors of cardiovascular diseases.

### 2.1. Medication Prescription

**Figure 1:**
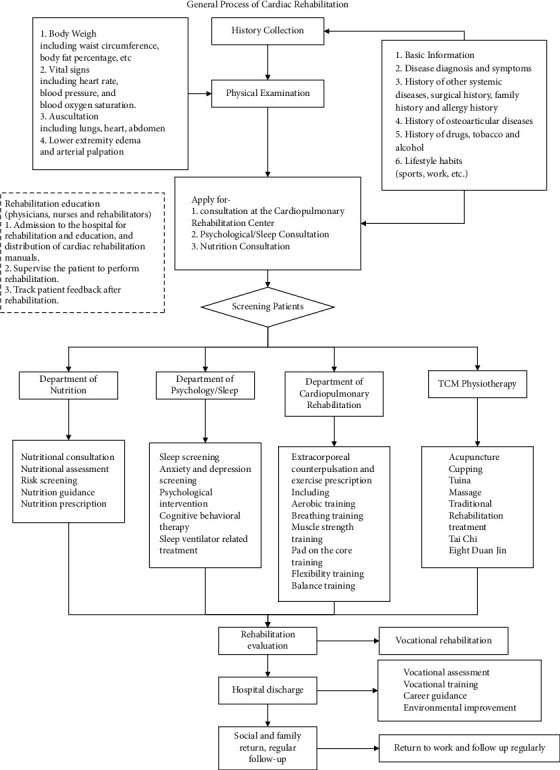
General process of cardiac rehabilitation. The flowchart summarized the standard procedure of cardiac rehabilitation for patients in China.

Medication and surgery (including interventional therapy) are important modalities in the treatment of cardiovascular diseases. Both can ameliorate and stabilize most diseases within a short period and provide an important foundation for further long-term cardiac rehabilitation. Most patients under cardiac rehabilitation have chronic cardiovascular diseases and require long-term medication, which is another reason that medication prescription plays a basic role in cardiac rehabilitation. Certain guidelines should be followed during prescription. Currently, drugs with sufficient evidence for treatment of cardiovascular diseases include antiplatelet drugs, beta blockers, angiotensin-converting enzyme inhibitors (ACEI), angiotensin II receptor blockers (ARB), and statins [4]. Prescriptions are not invariable but should be dynamically adjusted under the guidance of specialists to achieve the best therapeutic effect. Overall, the general process of cardiac rehabilitation requires “individual therapy” which depends on multiple factors, including medical history, demographic information, and lifestyle. After screening the patients, detailed management of the patients who need cardiac rehabilitation includes nutritional prescription, psychological intervention, extracorporeal counterpulsation, and traditional Chinese medicine (TCM). A detailed description of the process of cardiac rehabilitation is shown in Figure 1.

### 2.2. Exercise Prescription

Exercise prescription plays a key role in various methods of cardiac rehabilitation and is also the determining factor of treatment success or failure. It involves various aspects of exercise: mode, frequency, intensity, and venue. At present, the major modes of exercise are aerobic training, dynamic and static resistance training, flexibility training, and balance training, among others [4].

Individualized exercise prescription guarantees exercise safety and effectiveness but is a difficult aspect of cardiac rehabilitation. However, there are no clear guidelines on creating exercise prescriptions. Presently, exploring various exercise modalities is the main direction that scholars focus on [5].

While the pathophysiological effects of exercise on the human body are still under continuous investigation, its benefits have been confirmed. Furthermore, most scholars believe that more advantages are seen with high-intensity intermittent exercise [6]. Experiments and clinical studies have proven that exercise provides benefits by increasing the active substances of vascular endothelial cells. These substances regulate blood vessels, enhance vagus nerve tension, and lower the level of plasma norepinephrine, among other mechanisms [4]. This in turn improves cardiac function, regulates blood lipid levels, lowers blood pressure, improves coronary artery and cerebral ischemia, and reduces the incidence of arrhythmia, among other benefits.

Exercise enhances cardiac function since it is conducive to improved myocardial remodeling [7]. The possible mechanisms responsible include reversal of the metabolic decoupling process and reduction of glucose uptake in patients with metabolic syndrome [8]. Angiogenesis in exercising muscles is mediated by the effect of vascular endothelial growth factor and platelet-derived growth factor on *β*-adrenergic receptors [2]. These processes are stimulated by insulin-like growth factor-1, which is expressed in proportion to exercise. In addition, animal models have proven that insulin-like growth factor-1 reverses adrenergic-related myocardial remodeling [9]. Recent studies have shown that exercise-related post-transcriptional genes can reduce myocardial remodeling through mRNA regulation of the interactions among metabolism, contraction, and epigenetic genes [10]. The increase in metabolic demand during exercise leads to an increase in mitochondrial division and changes in energy pathways within organelles [11]. The increase in mitochondrial content in muscles promotes the preferential oxidation of fat rather than carbohydrate, thus reducing the production of lactic acid and enhancing organ function [2].

Numerous studies have proven that exercise increases patients' 6-minute walking distance, prolongs the exercise time in cardiopulmonary exercise test, and significantly reduces the readmission rate, incidence of cardiovascular events, and mortality rate [2]. Another study has shown that exercise significantly improves exercise duration, peak oxygen consumption, and ventilator threshold [12]. In patients with heart failure with preserved ejection fraction, exercise significantly increased peak oxygen consumption, peak left ventricular ejection fraction, peak stroke volume, and peak cardiac output. This effect can be attributed to the enhanced oxidation efficiency of peripheral skeletal muscles, which in turn lead to improved ventricular-vascular coupling [2]. The 2016 European Society of Cardiology (ESC) Guidelines for the Diagnosis and Treatment of Acute and Chronic Heart Failure recommend that patients with chronic heart failure should actively carry out exercise-based cardiac rehabilitation [13].

Exercise can regulate nerve function, inhibit sympathetic tension, and excite parasympathetic nerves through reduction of aldosterone secretion and inhibition of sympathetic nerve excitability [14]. In addition, exercise inhibits the secretion of norepinephrine and endothelin-1 and improves endothelial function through the interactions between plasma adrenomedullin and atrial/brain natriuretic peptide, the latter being closely related to aerobic consumption [15]. Autonomic nervous system dysfunction may result in coronary artery contraction, increased myocardial oxygen consumption, and fatal cardiovascular events. All of these play a significant pathophysiological role in the early stages of myocardial infarction, essential hypertension, and chronic heart failure [16, 17]. On the other hand, hyperfunction of the sympathetic nervous system is a trigger for arrhythmia and sudden death. High-intensity exercise can effectively achieve autonomic nerve rebalancing in patients with chronic heart failure [17]. A large number of studies have shown that exercise reduces the incidence of myocardial infarction. Moreover, the same studies report improvements in oxygen carrying capacity, endothelial function, and health-related quality of life with high-intensity exercise. Patients with coronary heart disease, especially those with left ventricular dysfunction, are at high exercise risk. However, the risk stratification of exercise and prevention strategies require further study [2]. Previous studies have also shown that different types of exercise have positive effects on endothelial cell structure and endothelium-dependent relaxation (EDR) function. Exercise intervention can cause changes in endothelial-derived vasoactive substances, reduce the level of oxidative stress and inflammatory response, and also lead to a gradual increase in arterial fluid shear stress, which is quite beneficial to the structural remodeling of vascular endothelial cells [18, 19]. Exercise further increases the level of myocardial enzymes secreting angiogenesis-promoting follistatin-like protein 1 (FSTL1), which promotes endothelial cell proliferation and angiogenesis [20]. In recent years, studies have also shown a potential link between the fluid shear force in the arterial tube and the morphological structure of mitochondria in endothelial cells. Trials have shown that all types of exercise are beneficial for correcting endothelial dysfunction, which can reduce mortality and morbidity in patients, and the effects of different types of exercise interventions are not significantly different [21, 22].

### 2.3. Psychological Prescription (including Sleep Management)

Psychological factors share a close relationship with the development, progression, and outcome of diseases. Poor mental state is not only a causative factor in cardiovascular diseases but also affects disease outcomes through negative feedback. A survey showed that among patients in the cardiology department, the detection rate of mental and psychological disorders in outpatients and inpatients was 55% and 50%, respectively. Moreover, almost all patients undergoing stent implantation had different degrees of psychological problems [18]. Exploring effective psychological intervention measures with concurrent integration into cardiac rehabilitation plans to improve rehabilitation quality is the current research focus of scholars. Rozanski et al. [4] pointed out that the development and progression of cardiovascular diseases are related to anxiety, depression, certain personality traits, social isolation, and chronic life stress. The psychological changes in spouses, other relatives, and friends after cardiovascular events also impact the patients.

Psychological intervention aims to relieve tension, improve treatment compliance, and boost self-confidence. However, repetition is required to avoid the disadvantages of the simple biomedical model. Depressive symptoms were found to decrease from 17% to 6% after cardiac rehabilitation training. In addition, the mortality rate of patients with depression who completed rehabilitation treatment was 73% lower than those who did not [2]. Sleep quality is another factor closely related to mental state. A meta-analysis found that among 173,301 participants, in comparison to those who slept 7-8 h a day, those who received 6-7 h of sleep a day had an increased risk of hypertension by 7% while those who received less than 6 h sleep a day had an increased risk of hypertension by 35%. Furthermore, patients in the chronic sleep deprivation state of passive sleep insufficiency may show blood pressure elevation in the short term and cardiovascular risk factors such as systemic inflammatory response and impaired glucose tolerance in the long term [4].

### 2.4. Smoking Cessation Prescription

Smoking is an independent risk factor for cardiovascular diseases. Several studies have outlined the significant causative effect of smoking on coronary heart disease, atherosclerotic peripheral vascular diseases, and stroke. Smoking cessation can reduce the risk of cardiovascular disease morbidity and mortality, and its long-term benefits are at least equivalent to those of commonly used secondary preventive drugs for coronary heart disease such as aspirin and statins. Therefore, medical professionals must explain the negative effects of smoking and the benefits of smoking cessation to the patients and their families. The optimal treatment plan for tobacco dependence is a comprehensive method combining drug therapy, psychotherapy, and behavioral therapy.

### 2.5. Nutrition Prescription

Existing evidence-based medicine has proven that excessive intake of energy, saturated fat, and cholesterol and insufficient intake of vegetables and fruits increase the risk for cardiovascular diseases. Conversely, the intake of a scientific and reasonable diet was found to reduce this risk. Considering this, the purpose of nutrition prescription is to guide patients in forming healthy eating habits.

The implementation of nutrition prescription is crucial to effective cardiac rehabilitation. Rehabilitation evaluation is an important starting point in the rehabilitation process. Current disease condition, exercise ability, nutrition, and psychological status of patients can be comprehended through rehabilitation evaluation. This enables the formulation of individualized, safe, and accurate rehabilitation plans. In America and developed European countries, the cardiopulmonary exercise test is considered the “gold standard” for clinical evaluation of cardiopulmonary function and formulation of exercise prescription [5]. All patients under cardiac rehabilitation must undergo pre-rehabilitation evaluation, rehabilitation process evaluation, and rehabilitation stage evaluation. Lastly, timely corrections must be made in prescription deviations.

## 3. Modes of Cardiac Rehabilitation

The current focus of research is to explore cardiac rehabilitation modalities that offer easy facilitation and high patient compliance while ensuring safe and effective rehabilitation. However, rehabilitation should be conducted under the guidance of cardiologists regardless of the chosen modality.

Cardiac rehabilitation can be classified into three modes according to location of implementation: inpatient rehabilitation (stage I rehabilitation), early outpatient rehabilitation or clinic rehabilitation (stage II rehabilitation), and long-term outpatient rehabilitation (stage III rehabilitation). These modes correspond to high-risk, medium-risk, and low-risk patients, respectively, with respect to disease condition.

Stage I rehabilitation refers to cardiac rehabilitation under the guidance and supervision of medical staff in the hospital. The goals of this mode include shortening the length of hospital stay and promoting the recovery of patients' daily life and exercise ability while avoiding the adverse effects caused by bed rest (such as reduced exercise tolerance and thromboembolic complications). In addition, stage I also involves reminding patients of smoking cessation and providing comprehensive and complete information in preparation for stage II rehabilitation. It is generally implemented in high-risk patients. Stage II rehabilitation is generally carried out in the first 6 months after discharge. Patients continue to undergo rehabilitation treatment under the guidance and supervision of medical staff at the outpatient clinic. At the onset of disease stabilization, self-monitored exercises may be carried out at home, and remote network and electronic information technology can be used to supervise the entire process of their cardiac rehabilitation. Generally, it is adopted for medium-risk patients. Stage III rehabilitation involves long-term rehabilitation at home. Patients undergo in-hospital evaluation, and rehabilitation exercise is carried out at home under the guidance of rehabilitation personnel and self-management to cultivate a healthy lifestyle. It is generally utilized in low-risk patients [19–22].

Selection of rehabilitation mode is influenced by factors such as income, education level, and the establishment level of tiered medical services. The United States implements hospital-based cardiac rehabilitation mode, with stage I rehabilitation being paid by medical insurance. Europe implements the mode of rehabilitation center combined with community, while Taiwan in China and Japan adopt the mode of family-driven rehabilitation clinic. In the Chinese mainland, the hospital cardiac rehabilitation mode, the family cardiac rehabilitation mode, or a mixture of both has begun to play a role in community cardiac rehabilitation [4]. Most of the cardiovascular treatment levels in China's tertiary hospitals have been in line with international standards. Among them, stage I rehabilitation after interventional surgery, that is, the hospital cardiac rehabilitation model, has been widely used in clinical practice with good results. The family cardiac rehabilitation model lacks a unified management model. It is generally guided by indirect forms such as self-help education and rehabilitation manuals, telephone supervision, and return visits, and it is difficult to grasp the relevant risk factors outside the hospital [4].

Another difficulty in cardiac rehabilitation is improving patient compliance [23, 24]. Studies have reported that only 15%–50% of patients under cardiac rehabilitation could continue rehabilitation for 6 months after the end of treatment, and even fewer patients could continue for 12 months [25]. At 6 months after the onset of the cardiovascular event, approximately 50% of smokers had quit smoking, and less than 50% of obese patients could comply to dietary recommendations [26]. AHA encourages nurses or non-medical care personnel to take the roles of the main supervisors and managers of family and community rehabilitation to increase the participation rate [18]. A prospective, multi-center, controlled study showed that strengthening follow-up after rehabilitation could achieve positive results, with high feasibility and satisfaction [27]. The rapid development of the Internet has seen application of more technologies in cardiac rehabilitation with beneficial results. Lunde et al. [28] conducted a single-group pre-test and post-test control study lasting 12 weeks, wherein patients utilized an application to help guide changes or maintain a healthy lifestyle. The results of this study show that the follow-up intervention based on this technology not only resulted in patient benefit but also stimulated their motivation for rehabilitation with high satisfaction. In another study, the use of a mobile application to monitor patients taking ticagrelor post-myocardial infarction showed significantly improved medication compliance [29]. A study by Gabelhousej et al. [30] showed that the hospital-centered, community-based combined cardiac rehabilitation mode integrating mobile technology had a similar effect as traditional cardiac rehabilitation mode but had the advantage of convenience. Research has also explored the application of virtual reality (VR) in the field of cardiac rehabilitation [31]. Strong evidence has led to the assumption that technologies such as artificial intelligence and the Internet will play a significant role in the field of cardiac rehabilitation in future.

## 4. Conclusions

Presently, cardiac rehabilitation has been internationally recognized as a safe, effective, and cost-effective method for treating patients with cardiovascular diseases. Research on cardiac rehabilitation has shifted from the demonstration of various mechanisms and principles to the exploration of various patient-centered rehabilitation methods and modes. However, there is still a lack of an individualized and standardized cardiac rehabilitation mode with high patient compliance. In addition, the large-scale promotion of modes such as telerehabilitation, community rehabilitation, and family rehabilitation still requires further research and demonstration. The application of modern technologies such as the Internet and artificial intelligence in cardiac rehabilitation will actively promote the development of cardiac rehabilitation.

## Data Availability

The data that support the findings of this study are available from the corresponding author upon reasonable request.

## References

[1] Supervia M., Turk-Adawi K., Lopez-Jimenez F. (2019). Nature of cardiac rehabilitation around the globe. *EClinicalMedicine*.

[2] Li X., Peng J. (2021). Research advances in cardiac rehabilitation and cardiovascular disease. *Chinese Journal of Gerontology*.

[3] Thomas R. J., King M., Lui K., Oldridge N., Pina I. L., Spertus J. (2007). AACVPR/ACC/AHA 2007 performance measures on cardiac rehabilitation for referral to and delivery of cardiac rehabilitation/secondary prevention services. *Circulation*.

[4] Gai S., Lu T., Xu H., Wei H., Lu Y. (2021). A review of the basic connotations of the concept of cardiac rehabilitation. *Chinese Journal of Cardiovascular Rehabilitation*.

[5] Chen Q., Yang F., Lu D. (2020). A hot topic of cardiac rehabilitation research based on pubmed database and co-word clustering analysis. *South China Journal of Cardiovascular Diseases*.

[6] Rognmo Ø, Moholdt T., Bakken H. (2012). Cardiovascular risk of high-versus moderate-intensity aerobic exercise in coronary heart disease patients. *Circulation*.

[7] Wilson M. G., Ellison G. M., Cable N. T. (2016). Basic science behind the cardiovascular benefits of exercise. *British Journal of Sports Medicine*.

[8] Schaun M. I., Marschner R. A., Peres T. R., Markoski M. M., Lehnen A. M. (2017). Aerobic training prior to myocardial infarction increases cardiac GLUT4 and partially preserves heart function in spontaneously hypertensive rats. *Applied Physiology Nutrition and Metabolism*.

[9] Roof S. R., Boslett J., Russell D. (2016). Insulin-like growth factor 1 prevents diastolic and systolic dysfunction associated with cardiomyopathy and preserves adrenergic sensitivity. *Acta Physiologica*.

[10] Soci U. P. R., Fernandes T., Barauna V. G. (2016). Epigenetic control of exercise training-induced cardiac hypertrophy by miR-208. *Clinical Science*.

[11] MacInnis M. J., Gibala M. J. (2017). Physiological adaptations to interval training and the role of exercise intensity. *Journal of Physiology*.

[12] Acanfora D., Scicchitano P., Casucci G. (2016). Exercise training effects on elderly and middle-age patients with chronic heart failure after acute decompensation: a randomized, controlled trial. *International Journal of Cardiology*.

[13] Ponikowski P., Voors A. A., Anker S. D. (2016). 2016 ESC guidelines for the diagnosis and treatment of acute and chronic heart failure. *Revista Española de Cardiología*.

[14] Szymanowicz L. D., Chmielewska M. F., Ratkowski W., Raczak G. (2013). Effect of various forms of physical training on the autonomic nervous system activity in patients with acute myocardial infarction. *Kardiologia Polska*.

[15] Krzeminski K. (2016). The role of adrenomedullin in cardiovascular response to exercise-a review. *Journal of Human Kinetics*.

[16] Esler M., Lambert E., Schlaich M. (2010). Point: chronic activation of the sympathetic nervous system is the dominant contributor to systemic hypertension. *Journal of Applied Physiology*.

[17] Wang J., Huang Y. (2020). Research progress of high-intensity interval training in the field of cardiac rehabilitation. *Advances in Cardiovascular Diseases*.

[18] Li C., Zuo A., Li T., Guo Y. (2021). Visual analysis of cardiac rehabilitation research hotspots based on web of science database. *Chinese General Practice*.

[19] Ding R., Lei S. (2021). The development process, status quo and thinking of cardiac rehabilitation in China. *Practical Journal of Cardiac Cerebral Pneumal and Vascular Disease*.

[20] Committee of Cardiac Rehabilitation and Prevention of Chinese Association of Rehabilitation Medicine (2021). China expert consensus on center guided home-based cardiac rehabilitation. *Zhonghua Nei Ke Za Zhi*.

[21] Han Q., Kuang J., Du H. (2020). The current situation of cardiac rehabilitation development in China. *Practical Journal of Cardiac Cerebral Pneumal and Vascular Disease*.

[22] Zhao S., Gao H. (2020). Research progress on the role of physical rehabilitation in coronary microcirculation revascularization. *Chinese Journal of Geriatrics*.

[23] Chen X., Jiang W., Lin X. (2018). Effect of an exercise-based cardiac rehabilitation program “Baduanjin Eight-Silken-Movements with self-efficacy building” for heart failure (BESMILE-HF study): study protocol for a randomized controlled trial. *Trials*.

[24] Ades P. A., Keteyian S. J., Wright J. S. (2017). Increasing cardiac rehabilitation participation from 20% to 70%: a road map from the million hearts cardiac rehabilitation collaborative. *Mayo Clinic Proceedings*.

[25] Tang Y., Huang S., Zhang C. (2021). Research advances in cardiac rehabilitation. *Trauma and Acute Critical Illness Medicine*.

[26] Kotseva K., Wood D., De Bacquer D. (2016). Euroaspire IV: a European Society of Cardiology survey on the lifestyle, risk factor and therapeutic management of coronary patients from 24 European countries. *European Journal of Preventive Cardiology*.

[27] Deck R., Beitz S., Baumbach C., Brunner S., Hoberg E., Knoglinger E. (2020). Rehab aftercare “new credo” in the cardiac follow-up rehabilitation. *Rehabilitation*.

[28] Lunde P., Nilsson B. B., Bergland A., Bye A. (2019). Feasibility of a mobile phone app to promote adherence to a heart-healthy lifestyle: single-arm study. *JMIR Formative Research*.

[29] Johnston N., Bodegard J., Jerstrom S. (2016). Effects of interactive patient smartphone support app on drug adherence and lifestyle changes in myocardial infarction patients: a randomized study. *American Heart Journal*.

[30] Gabelhousej A., Evesna B., Gracesld E., Reid R. C., Caperchione C. M. (2018). Traditional versus hybrid outpatient cardiac rehabilitation: a comparison of patient outcomes. *Journal of Cardiopulmonary Rehabilitation and Prevention*.

[31] Zhou Y., Zheng Y., Yan G., Chen J., Huang S. (2021). Research progress of virtual reality technology in the field of cardiac rehabilitation. *Jiangsu Medicine*.

